# Unraveling the nature of nano-diamonds and silica in a catheterized tapered artery: highlights into hydrophilic traits

**DOI:** 10.1038/s41598-023-32604-6

**Published:** 2023-04-07

**Authors:** Sara I. Abdelsalam, M. M. Bhatti

**Affiliations:** 1 Basic Science, Faculty of Engineering, The British University in Egypt, Al-Shorouk City, Cairo, 11837 Egypt; 2 College of Mathematics and Systems Science, Shandong University of Science and Technology, Qingdao, 266590 Shandong China

**Keywords:** Other nanotechnology, Applied mathematics

## Abstract

In this work, we observe the behavior of a hybrid nanofluidic model containing nanodiamonds and silica nanoparticles. The nanofluid propagates through a catheterized tapered artery with three distinct configurations: converging tapered, non-tapered and diverging tapered arteries. In order to assess the rheological properties of the blood, the third-grade non-Newtonian fluid is employed in the flow model such that the Newtonian versus non-Newtonian effects are revealed. The system of equations governing the flow is modeled under magnetic field and with heat transfer, then solved in a closed form using the perturbation approach for the pertinent parameters. The interpretations of the physical variables of interest, such as the velocity, temperature and wall shear stress, are explained. The integration of diamonds and silica nanoparticles give rise to diverse of biological applications since they are used in the drug delivery and biological imaging in genetic materials due to their hydrophilic surfaces. The present mathematical analysis lays a solid foundation on possible therapeutic applications in biomedicine.

## Introduction

Diamond nanoparticles, often known as nanodiamonds (NDs), are particles with a size of less than 100 nm. They have a particle size that is thousands of times smaller than human hair^1^. Nanodiamonds are quickly gaining traction as viable carriers for several therapies through drug delivery since they have a customizable surface, high biocompatibility and a wide surface area that allows for the hybridization of compounds such as medicines and genes in extra- and intracellular delivery. Many studies have significantly contributed to the improvement of NDs by utilizing their diverse characteristics for biological purposes^2^. Due to the ability of NDs to shuttle drugs through the blood–brain barrier, Alawadi et al.^3^ successfully investigated the loading of amlodipine onto NDs opening the doors for the delivery of other drugs into the brain. Owing to the neuroprotective effects of the NDs, various neuroprotective remedies depend upon NDs such as those that treat Alzheimer’s and Parkinson’s diseases^4^. Li et al.^5^ shed some light on NDs being a member of nanocarbon family that can be used in biomedical and bioengineering applications. In 2016, Tasi et al.^6^ investigated the interaction of the mice blood as well as the impact of the surface modification of NDs in the optimization of ND-blood interaction. Their findings support the safety of NDs in organisms, as well as the potential of using them without affecting the physiological conditions of blood. Chen and Zhang^7^ presented some insights into important topics about NDs and their potential developments in various applications. It was seen that due to the diamond's exceptional mechanical strength, vertically aligned diamond nanostructures are predicted to outperform other forms of vertical nanostructures in applications requiring mechanical force. It has been noticed by Masys et al.^8^ that hydrogenated NDs have hydrophilic traits so that the molecules of water get attracted to particular sites at the surface. The hydrophilic characteristic was confirmed by Chauhan et al.^1^ where NDs were noticed to be excellent carriers of drugs and can be used in chronic disorders. In addition, it has been highlighted by Namdar and Nafisi^9^ that the nontoxicity of NDs along with their great potential in being absorbed through the skin due to their size make them a good choice for skin care products. The latter property has been taken to a higher level when some investigators researched the capability of NDs in treating superficial malignant tumors such the skin cancer, melanoma^10,11^.

Silica nanoparticles, SiNPs, are used to contain contrast agent particles such as organic dyes, gold nanoparticles and iron oxide, and they serve an essential auxiliary function in medical imaging. Nowadays, drug-delivery systems through red blood cells along with developed nanomedicines are attracting great interests of researchers^12,13^. It has been said that SiNPs are not hazardous up to a concentration of 100 mg/mL if the particle size is 100 nm as seen by Meng et al.^14^. They are further an excellent choice for controlled release of medicinal drugs thanks to their enormous surface area, pore capacity and great stability. There are natural sources to extract silica from such as sugarcane, coffee husk, rice husk, corn cob and much more^15^. Worth mentioning that SiNPs have been recently introduced in fighting SARS-Cov-2 by depositing silver nanocluster/silica on a mask to reduce infection to zero^16^. There are three types of SiNPs: solid, mesoporous and nonporous. Silica nanoparticles have become essential in the transport of medicines and genetic material due to their physical and chemical stability as well as their well-defined hydrophilic surface and the ability to shield pharmaceuticals from aggressive immune responses^17^. The hydrophilic nature of particles can also be utilized to alter the particles by surface adsorption of surfactants or short chain polymers via electrostatic attractions and hydrogen bonding^18^.

Blood, which is composed of plasma, white and red cells, may be considered one of nature's most essential multi-segment mixes. It can be considered as the third-grade non-Newtonian fluid^19^. In the latter work and with the use of the least squares approach, the authors studied the blood flow including nanoparticles via porous blood arteries in the presence of a magnetic field and heat transfer. Their results revealed that there is a huge change in the velocity distribution as a result of an increase in the Brownian and thermophoresis parameters. Parida and Padhy^20^ investigated numerically the third-grade fluid flow, representing the blood, in a porous conduit with heat transfer and uniform transverse magnetic field. They found out that the fluid temperature is enhanced as a result of a diffusion in heat within the conduit. In 2013, Baoku et al.^21^ researched the impact of heat and mass transfer along with magnetic effects and partial slip on a third-grade fluid flow in an infinite vertical insulated plate with suction along the boundary layer. It was seen that when the porous plate is insulated, the magnetic field strength is reduced with an enhanced temperature. Sobamowo et al.^22^ used the Runge–Kutta Fehlberg procedure and shooting method to discuss the impact of thermal radiation and magnetic field of a third-grade fluid between two disks in a porous space. It was concluded that the rate of heat transfer is increased as a result to an increase in the radiation parameter. Rahbari et al.^23^ considered the blood as a third-grade fluid and investigated the inclusion of nanoparticles within the flow with magnetic effect through a porous artery. They discovered that the nanoparticles concentration increases as a result of an increase in the thermophoresis number. For more information about the inclusion of non-Newtonian fluids with magnetic effect and/or thermal effect through various physiological applications, readers are referred to Refs.^24–27^ and to the references therein.

Taking the aforementioned considerations and restrictions into account, we intend to study the effects arising from adding diamonds and silica nanoparticles to a flow field where blood is represented with a third-grade fluid. The flow is inspected through a catheterized tapered artery with three distinct configurations: converging tapered, non-tapered and diverging tapered arteries. For more tackled biological applications, we take the magnetic field and heat transfer into considerations and test the impact of all pertinent parameters on the flow rheology. Since the hybridity of both nanoparticles, diamonds and silica, has not been investigated, according to our knowledge, we wish to harness such nanoparticles in investigating their impact when incorporated in various types of conduits. It is expected that the current study will aid in developing future nanoscale drug carriers in biological systems.

## Mathematical and physical modeling of the blood flow

**Figure 1:**
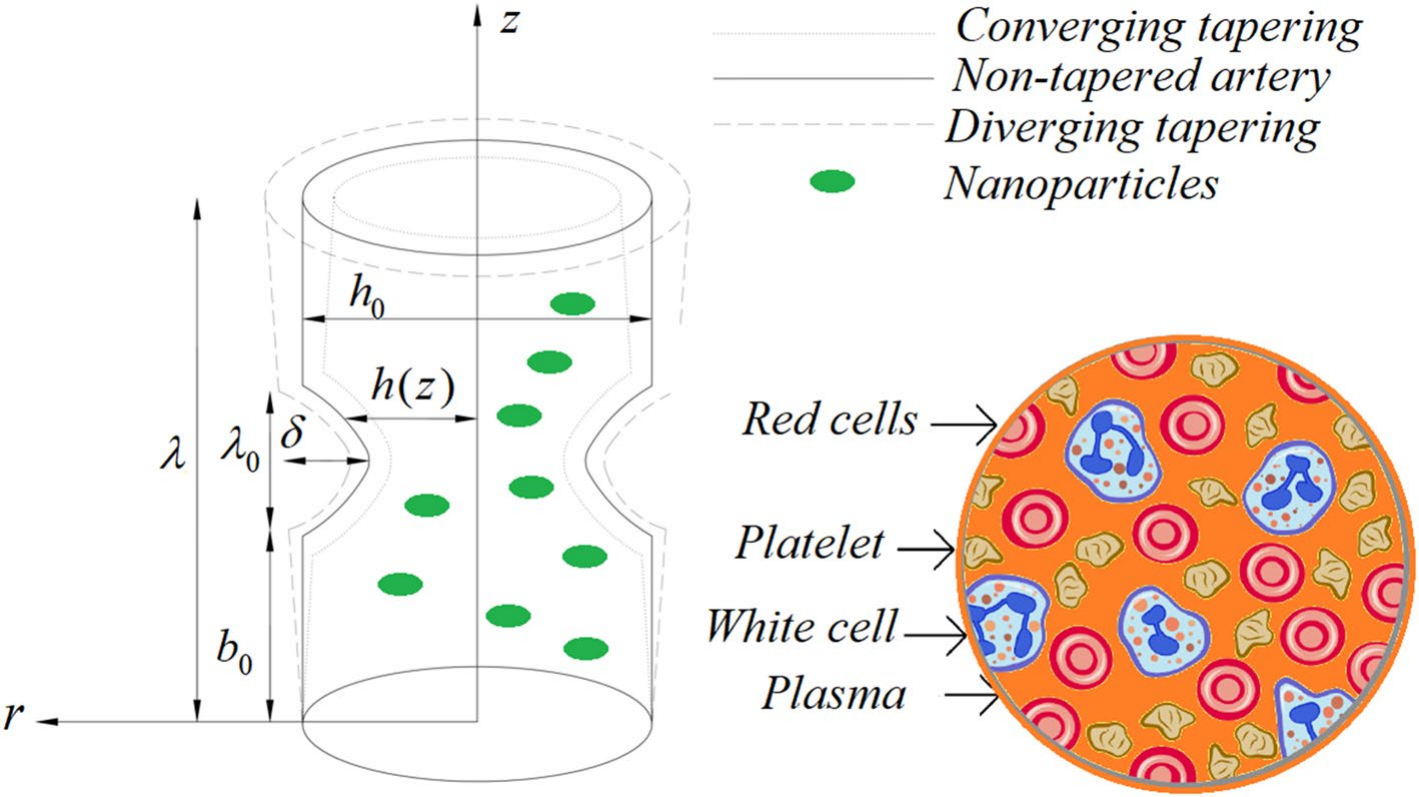
Simplified configuration of blood flow through the tapered artery and their composition in the presence of nanoparticles.

Consider a catheterized tapered artery having length $\lambda$ filled with hybrid third-grade nanofluid in the presence of diamond and silica nanoparticles. The third-grade nanofluid is considered as blood with incompressible and irrotational features. The third-grade nanofluid is electrically conducting due to the strong influence of an extrinsic magnetic field. The system of cylindrical polar coordinates $\left( {r,\theta ,z} \right)$ is considered, whereas *r* is located in the radial direction, *z* is located along the flow direction and $\theta$ is located along the circumferential direction. Furthermore, the third-grade nanofluid propagates in the presence of heat transfer phenomena where $\tilde{T}_{{{\text{ref}}}}$ is the temperature at the artery and $\tilde{T}_{1}$ is the temperature at the endoscopic wall. The mathematical form of the proposed geometry is described as:

1 $$ h(z) = \left\{ \begin{gathered} \left( {h_{0} + z\xi } \right)\left[ {1 - \frac{{n^{{\frac{n}{n - 1}}} \delta }}{{\lambda_{0}^{n} h_{0} \left( {n - 1} \right)}}\left\{ {\lambda_{0}^{n - 1} \left( {z - b_{0} } \right) - \left( {z - b_{0} } \right)^{n} } \right\}} \right],\quad b_{0} < z \le b_{0} \lambda_{0} , \hfill \\ \left( {h_{0} + z\xi } \right)\quad \quad \quad \quad \quad \quad \quad \quad \quad \quad \quad \quad \quad \quad \quad \quad \quad \quad \quad \quad \quad \quad \quad {\text{otherwise}} \hfill \\ \end{gathered} \right. $$

where $\delta$ represents the height of the stenosis which is situated at $z = b_{0} + \frac{{\lambda_{0} }}{{n^{{\left( {n - 1} \right)/n}} }}$, $h_{0}$ is the radius of the non-tapered artery in a non-stenotic part, $\xi = \tan \psi$ is the tapering parameter, i.e., $\psi$ is the tapering angle where non-tapered artery is at $\psi = 0$, diverging tapered artery is at $\psi > 0$ and converging tapered artery at is at $\psi < 0$. Also, $\lambda_{0}$ is the length of the stenosis, $n \ge 2$ represents the configuration of the constriction profile which belongs to shape parameter in which symmetric stenosis can be obtained for $n = 2$, and $b_{0}$ shows the location of the stenosis as shown in Fig. 1.

The equations governing the flow model can then be written as

2 $$ \nabla \cdot {\tilde{\mathbf{V}}} = 0, $$

3 $$ \rho_{hnf} \left( {\frac{{\partial {\tilde{\mathbf{V}}}}}{\partial t} + {\tilde{\mathbf{V}}} \cdot \nabla {\tilde{\mathbf{V}}}} \right) = \nabla \cdot \overline{\xi } + {\text{J}} \times {\text{B}} + \left( {\rho \beta } \right)_{hnf} g\left( {\tilde{T} - \tilde{T}_{{{\text{ref}}}} } \right), $$

where ${\tilde{\mathbf{V}}}$ has the components of velocity, ${\text{B}}$ is the magnetic field, ${\text{J}}$ is the current density, ${\text{J}} \times {\text{B}} = - \sigma_{hnf} B_{0}^{2} {\tilde{\mathbf{V}}}$, $\sigma_{hnf}$ is the electrical conductivity of hybrid nanofluid, $B_{0}$ is the applied magnetic field, $\beta_{hnf}$ denotes the thermal expansion coefficient, $\tilde{T}$ is the nanofluid temperature, $g$ the accelaration due to gravity, $T_{{{\text{ref}}}}$ is the reference temperature, $\rho_{hnf}$ represents the density of hybrid nanofluid, $k$ is the porosity parameter, $\mu_{hnf}$ is the viscosity of the hybrid nanofluid, the subscript $hnf$ represents the hybrid nanofluid and $\overline{\xi }$ is the stress tensor for the third-grade fluid model and is defined as:

4 $$ \overline{\xi } = - p{\text{I + }}\mu_{hnf} D_{1} + \alpha_{1} D_{2} + \alpha_{2} D_{1}^{2} + \beta_{1} D_{3} + \beta_{1} \left[ {D_{1} D_{2} + D_{2} D_{1} } \right] + \beta_{3} \left( {{\text{tr}}D_{1}^{2} } \right)D_{1} , $$

where $p{\text{I}}$ denotes the pressure, $\mu_{hnf}$ is the viscosity coefficient, $\left( {\alpha_{n} ,\beta_{n} } \right)$’*s* is the material moduli, ${D}_{1},{D}_{2}$ and ${D}_{3}$ are the first three Rivlin-Erickson tensors that are described as

5 $$ D_{1} = G + G^{T} ,\;{\text{and}}\;D_{m} = \dot{D}_{m - 1} + D_{m - 1} G + G^{T} D_{m - 1} ,\quad m = 2,3 $$

where $G=grad(v)$, $v$ is the velocity components, $T$ in superscript denotes the transpose of the matrix and the dot represents the material time differentiation.

The temperature equation under the presence of viscous dissipation and joule heating reads as

6 $$ \left( {\frac{{\partial \tilde{T}}}{\partial t} + {\tilde{\mathbf{V}}} \cdot \nabla \tilde{T}} \right) = \frac{{\kappa_{hnf} }}{{\left( {\rho C_{p} } \right)_{hnf} }}\nabla \cdot \left( {\nabla \tilde{T}} \right) + \frac{{{\text{J}} \cdot {\text{J}}}}{{\left( {\rho C_{p} } \right)_{hnf} \sigma_{hnf} }} + \frac{{\overline{\xi }}}{{\left( {\rho C_{p} } \right)_{hnf} }} \cdot \nabla {\tilde{\mathbf{V}}}, $$

where $\kappa_{hnf}$ and $\left( {\rho C_{p} } \right)_{hnf}$ characterize the thermal conductivity and the heat capacity of the hybrid nanofluid, respectively. The thermo-physical properties of density, heat capacity, dynamic viscosity, thermal conductivity, thermal expansion coefficient and electric conductivity are defined in the following equations:

i Density:
  7 $$ \begin{gathered} \rho_{nf} = \rho_{f} \left[ {1 - \Phi_{1} } \right] + \Phi_{1} \rho_{np1} , \hfill \\ \rho_{hnf} = \left[ {1 - \Phi_{2} } \right]\left\{ {\rho_{f} \left[ {1 - \Phi_{1} } \right] + \Phi_{1} \rho_{np1} } \right\}, \hfill \\ \end{gathered} $$
ii Heat capacity:
  8 $$ \begin{gathered} \left( {\rho C_{p} } \right)_{nf} = \left[ {1 - \Phi_{1} } \right]\left( {\rho C_{p} } \right)_{f} + \Phi_{1} \left( {\rho C_{p} } \right)_{np1} , \hfill \\ \left( {\rho C_{p} } \right)_{hnf} = \left[ {1 - \Phi_{2} } \right]\left\{ {\left[ {1 - \Phi_{1} } \right]\left( {\rho C_{p} } \right)_{f} + \Phi_{1} \left( {\rho C_{p} } \right)_{np1} } \right\}\Phi_{2} \left( {\rho C_{p} } \right)_{np2} . \hfill \\ \end{gathered} $$
iii Dynamic viscosity:
  9 $$ \begin{gathered} \mu_{nf} = \frac{{\mu_{f} }}{{\left[ {1 - \Phi_{1} } \right]^{2.5} }}, \hfill \\ \mu_{hnf} = \frac{{\mu_{f} }}{{\left[ {1 - \Phi_{1} } \right]^{2.5} \left[ {1 - \Phi_{2} } \right]^{2.5} }}. \hfill \\ \end{gathered} $$
iv Thermal conductivity:
  10 $$ \begin{gathered} \kappa_{nf} = \kappa_{f} \times \left[ {\frac{{\kappa_{np1} + 2\kappa_{f} - 2\Phi_{1} \left( {\kappa_{f} - \kappa_{np1} } \right)}}{{\kappa_{np1} + 2\kappa_{f} + \Phi_{1} \left( {\kappa_{f} - \kappa_{np1} } \right)}}} \right], \hfill \\ \kappa_{hnf} = \kappa_{nf} \times \left[ {\frac{{\kappa_{np2} + 2\kappa_{nf} - 2\Phi_{2} \left( {\kappa_{nf} - \kappa_{np2} } \right)}}{{\kappa_{np2} + 2\kappa_{nf} + \Phi_{2} \left( {\kappa_{nf} - \kappa_{np2} } \right)}}} \right]. \hfill \\ \end{gathered} $$
v Thermal expansion coefficient:
  11 $$ \begin{gathered} \left( {\rho \beta } \right)_{nf} = \left[ {1 - \Phi_{1} } \right]\left( {\rho \beta } \right)_{f} + \Phi_{1} \left( {\rho \beta } \right)_{np1} , \hfill \\ \left( {\rho \beta } \right)_{hnf} = \left[ {1 - \Phi_{2} } \right]\left\{ {\left[ {1 - \Phi_{1} } \right]\left( {\rho \beta } \right)_{f} + \Phi_{1} \left( {\rho \beta } \right)_{np1} } \right\} + \Phi_{2} \left( {\rho \beta } \right)_{np2} . \hfill \\ \end{gathered} $$
vi Electric conductivity:
  12 $$ \begin{gathered} \sigma_{nf} = \sigma_{f} \times \left[ {\frac{{\sigma_{np1} \left( {1 + 2\Phi_{1} } \right) + 2\sigma_{f} \left( {1 - \Phi_{1} } \right)}}{{\sigma_{np1} \left( {1 - \Phi_{1} } \right) + \sigma_{f} \left( {2 + \Phi_{1} } \right)}}} \right], \hfill \\ \sigma_{hnf} = \sigma_{nf} \times \left[ {\frac{{\sigma_{np2} \left( {1 + 2\Phi_{2} } \right) + 2\sigma_{f} \left( {1 - \Phi_{2} } \right)}}{{\sigma_{np2} \left( {1 - \Phi_{2} } \right) + \sigma_{f} \left( {2 + \Phi_{2} } \right)}}} \right]. \hfill \\ \end{gathered} $$
  where ${\Phi }_{1}$ and ${\Phi }_{2}$ represent the nanoparticle volume fraction, and $np1$ and $np2$ represent the nanoparticles of diamond and silica, respectively.

## Lubrication approach

The proposed governing equation are modelled using the lubrication approach. Therefore, in order to obtain the proposed model in the dimensionless form and the scaled variables are defined as:

13 $$ \begin{gathered} \tilde{r} = \frac{r}{{R_{0} }},\;\tau = \frac{{\lambda_{0} \tau }}{{R_{0} }},\;\tilde{v} = \frac{{\lambda_{0} }}{{u_{{{\text{ave}}}} \delta }}v,\;\tilde{R} = \frac{R}{{R_{0} }},\;\tilde{p} = \frac{{R_{0}^{2} }}{{u_{{{\text{ave}}}} \lambda_{0} \mu }}p, \hfill \\ \tilde{z} = \frac{z}{{R_{0} }},\;T = \frac{{\tilde{T} - \tilde{T}_{{{\text{ref}}}} }}{{\tilde{T}_{1} - \tilde{T}_{{{\text{ref}}}} }},\;\tilde{\delta } = \frac{\delta }{{R_{0} }},\;\lambda = \frac{L}{{\lambda_{0} }},\;\tilde{u} = \frac{u}{{u_{{{\text{ave}}}} }}. \hfill \\ \end{gathered} $$

where $u_{{{\text{ave}}}}$ is the averaged velocity of the whole tube over a whole section. Using Eq. (13) into Eqs. (4–6), we obtain the dimensionless equations as (after removing the tilde):

14 $$ \frac{\partial p}{{\partial z}} = \frac{1}{r}\frac{\partial }{\partial r}\left[ {r\frac{\partial w}{{\partial r}}\left( {C_{1} + \zeta \left( {\frac{\partial w}{{\partial r}}} \right)^{2} } \right)} \right] - C_{2} M^{2} w + G_{r} C_{3} T, $$

15 $$ \frac{{C_{4} }}{r}\frac{\partial }{\partial r}\left( {r\frac{\partial T}{{\partial r}}} \right) + B_{m} C_{2} M^{2} w^{2} + B_{m} \left[ {C_{1} \left( {\frac{\partial u}{{\partial r}}} \right)^{2} + \zeta \left( {\frac{\partial u}{{\partial r}}} \right)^{4} } \right], $$

where $\zeta$ is the third-grade fluid parameter, *Ec* is the Eckert number, *M* is the Hartmann number, $Gr$ is the thermal Grashof number and Pr is the Prandtl number. They are defined as

16 $$ \begin{gathered} \zeta = \frac{{2u_{{{\text{ave}}}}^{2} \left( {\beta_{2} + \beta_{3} } \right)}}{{\mu_{f} R_{0}^{2} }},\;Ec = \frac{{u_{{{\text{ave}}}}^{2} }}{{\left( {\tilde{T}_{1} - \tilde{T}_{{{\text{ref}}}} } \right)\left( {C_{p} } \right)_{f} }},\;M = \sqrt {\frac{{\sigma_{f} }}{{\mu_{f} }}} B_{0} R_{0} , \hfill \\ \Pr = \frac{{\left( {\rho C_{p} } \right)_{f} \upsilon_{f} }}{{\kappa_{f} }},\;G_{r} = \frac{{\left( {\tilde{T}_{1} - \tilde{T}_{{{\text{ref}}}} } \right)\left( {\rho \beta } \right)_{f} gR_{0}^{2} }}{{\mu_{f} u_{{{\text{ave}}}} }},\;B_{m} = Ec \times \Pr . \hfill \\ \end{gathered} $$

Here, *B*_*m*_ is the Brinkman number and the reaming parameters ${C}_{1},{C}_{2},{C}_{3}$ and ${C}_{4}$ are defined as:

17 $$ C_{1} = \frac{{\mu_{hnf} }}{{\mu_{f} }},\;C_{2} = \frac{{\sigma_{hnf} }}{{\sigma_{f} }},\;C_{3} = \frac{{\left( {\rho \beta } \right)_{hnf} }}{{\left( {\rho \beta } \right)_{f} }},\;C_{4} = \frac{{\kappa_{hnf} }}{{\kappa_{f} }}, $$

The boundary conditions in dimensionless form are defined as:

18 $$ \left\{ \begin{gathered} w^{\prime} = T^{\prime} = 0\quad \quad {\text{at }}r = 0, \hfill \\ w = 0, T = 0 \, \quad {\text{at }}r = R. \hfill \\ \end{gathered} \right. $$

## Series solutions using perturbation approach

The formulated Eqs. (14–15) are nonlinear, therefore exact solutions are not possible for such cases. We will employ a perturbation approach to obtain the series solutions. More details on the proposed methodology is given in the references^28,29^. The perturbation scheme for the formulated equations are defined as

19 $$ \hbar \left( {\overline{w},\varepsilon } \right) = \left( {1 - \varepsilon } \right)\left[ {\Theta \left( {\overline{w}} \right) - \Theta \left( {w_{0} } \right)} \right] + \varepsilon \left[ {\Theta \left( {\overline{w}} \right) + \frac{\zeta }{{C_{1} r}}\left( {\frac{{\partial \overline{w}}}{\partial r}} \right)^{3} + \frac{\zeta }{{C_{1} }}\frac{\partial }{\partial r}\left( {\frac{{\partial \overline{w}}}{\partial r}} \right)^{3} - \frac{{C_{2} }}{{C_{1} }}M^{2} \overline{u} + G_{r} \frac{{C_{3} }}{{C_{1} }}\overline{T} - \frac{1}{{C_{1} }}\frac{dp}{{dz}}} \right], $$

20 $$ \hbar \left( {\overline{T},\varepsilon } \right) = \left( {1 - \varepsilon } \right)\left[ {\Theta \left( {\overline{T}} \right) - \Theta \left( {T_{0} } \right)} \right] + \varepsilon \left[ {\Theta \left( {\overline{T}} \right) + \frac{{B_{m} }}{{C_{4} }}\left( {C_{1} \left( {\frac{\partial u}{{\partial r}}} \right)^{2} + \zeta \left( {\frac{\partial u}{{\partial r}}} \right)^{4} } \right) + \frac{{C_{2} M^{2} B_{m} }}{{C_{4} }}\overline{w}^{2} } \right], $$

where $\varepsilon$ is the artificial embedded parameter.

The form of linear operator is chosen as

21 $$ \Theta = \frac{1}{r}\frac{\partial }{\partial r}\left( {r\frac{\partial }{\partial r}} \right). $$

In view of Eqs. (18) and (21), the appropriate initial guess is defined as

22 $$ w_{0} = T_{0} = \frac{{r^{2} - R^{2} }}{4}. $$

Defining the expansion

23 $$ \overline{w} = \overline{w}_{0} + \varepsilon \overline{w}_{1} + \varepsilon^{2} \overline{w}_{2} + \ldots , $$

24 $$ \overline{T} = \overline{T}_{0} + \varepsilon \overline{T}_{1} + \varepsilon^{2} \overline{T}_{2} + \ldots , $$

### Zeroth order system

At zeroth order, we obtain the following set of differential equations

25 $$ \Theta \left( {\overline{w}_{0} } \right) - \Theta \left( {w_{0} } \right) = 0, $$

26 $$ \Theta \left( {\overline{T}_{0} } \right) - \Theta \left( {T_{0} } \right) = 0, $$

The solutions of the latter equations can be written as

27 $$ \overline{w}_{0} = \overline{T}_{0} = \frac{{r^{2} - R^{2} }}{4}. $$

### First order system $\left( \varepsilon \right)$

At first order, we obtain the following set of differential equations

28 $$ \Theta \left( {\overline{w}_{1} } \right) + \Theta \left( {w_{0} } \right) + \frac{\zeta }{{C_{1} r}}\left( {\frac{{\partial \overline{w}_{0} }}{\partial r}} \right)^{3} + \frac{\zeta }{{C_{1} }}\frac{\partial }{\partial r}\left( {\frac{{\partial \overline{w}_{0} }}{\partial r}} \right)^{3} - \frac{{C_{2} }}{{C_{1} }}M^{2} \overline{w}_{0} + G_{r} \frac{{C_{3} }}{{C_{1} }}\overline{T}_{0} - \frac{1}{{C_{1} }}\frac{dp}{{dz}}, $$

29 $$ \Theta \left( {\overline{T}_{1} } \right) + \Theta \left( {T_{0} } \right) + \frac{{B_{m} }}{{C_{4} }}\left( {C_{1} \left( {\frac{{\partial \overline{w}_{0} }}{\partial r}} \right)^{2} + \zeta \left( {\frac{{\partial \overline{w}_{0} }}{\partial r}} \right)^{4} } \right) + \frac{{C_{2} M^{2} B_{m} }}{{C_{4} }}\overline{w}_{0}^{2} , $$

The solutions of the above equations can be written as

30 $$ \overline{w}_{1} = - \frac{{\left( {r^{2} - R^{2} } \right)\left( {16C_{1} - 16dp/dz + A3G_{r} r^{2} - C_{2} M^{2} r^{2} - 3C_{3} G_{r} R^{2} + 3C_{2} M^{2} R^{2} + 2r^{2} \zeta + 2R^{2} \zeta } \right)}}{{64C_{1} }}, $$

31 $$ \overline{T}_{1} = - \frac{{\left( {r^{2} - R^{2} } \right)\left[ {288C_{4} + B_{m} \left( {18C_{1} \left( {r^{2} + R^{2} } \right) + C_{2} M^{2} \left( {2r^{4} - 7r^{2} R^{2} + 11R^{4} } \right) + 2\left( {r^{4} + r^{2} R^{2} + R^{4} } \right)\zeta } \right)} \right]}}{{1152C_{4} }}, $$

### Second order system $\left( {\varepsilon^{2} } \right)$

At second order, we obtain the following set of differential equations

32 $$ \Theta \left( {\overline{w}_{2} } \right) + \frac{3\zeta }{{C_{1} r}}\left( {\frac{{\partial \overline{w}_{0} }}{\partial r}} \right)^{2} \left( {\frac{{\partial \overline{w}_{1} }}{\partial r}} \right) + \frac{\zeta }{{C_{1} }}\frac{\partial }{\partial r}\left\{ {\left( {\frac{{\partial \overline{w}_{1} }}{\partial r}} \right)\left( {\frac{{\partial \overline{w}_{0} }}{\partial r}} \right)^{2} } \right\} - \frac{{C_{2} }}{{C_{1} }}M^{2} \overline{w}_{1} + G_{r} \frac{{C_{3} }}{{C_{1} }}\overline{T}_{1} , $$

33 $$ \Theta \left( {\overline{T}_{2} } \right) + \frac{{B_{m} }}{{C_{4} }}\left( {2C_{1} \frac{{\partial \overline{w}_{0} }}{\partial r}\frac{{\partial \overline{w}_{1} }}{\partial r} + 4\zeta \left( {\frac{{\partial \overline{w}_{0} }}{\partial r}} \right)^{3} \frac{{\partial \overline{w}_{1} }}{\partial r}} \right) + \frac{{2C_{2} M^{2} B_{m} }}{{C_{4} }}\overline{w}_{1} \overline{w}_{0} . $$

The solutions of the latest equations can be written as

34 $$ \begin{gathered} \overline{w}_{2} = \frac{{\left( {r^{2} - R^{2} } \right)}}{{36864C_{1}^{2} C_{4} }}\left[ {16C_{1}^{2} C_{3} B_{m} G_{r} \left( {r^{4} + r^{2} R^{2} - 8R^{4} } \right) + 16C_{4} \left\{ {C_{2}^{2} M^{4} \left( {r^{4} - 8r^{2} R^{2} + 19R^{4} } \right)} \right.} \right. \hfill \\ \quad \quad \; + C_{2} M^{2} \left( {36dp/dz\left( {r^{2} - 3R^{2} } \right) - C_{3} G_{r} \left( {r^{4} - 8r^{2} R^{2} + 19R^{4} } \right) - 20r^{4} \zeta + 34r^{2} R^{2} \zeta + 52R^{4} \zeta } \right) \hfill \\ \quad \quad \; + 18\zeta \left. {\left( { - 12dp/dz\left( {r^{2} + R^{2} } \right) + C_{3} G_{r} \left( {r^{4} - 2r^{2} R^{2} - 2R^{4} } \right) + 2\left( {r^{4} + r^{2} R^{2} + R^{4} } \right)\zeta } \right)} \right\} \hfill \\ \quad \quad { + }C_{1} \left\{ { - 576C_{4} \left( {C_{2} M^{2} \left( {r^{2} - 3R^{2} } \right) - 6\left( {r^{2} + R^{2} } \right)\zeta } \right) + C_{3} G_{r} \left\{ {576C_{4} \left( {r^{2} - 3R^{2} } \right)} \right.} \right. \hfill \\ \left. {\left. {\left. {\quad \quad \; + B_{m} \left( {C_{2} M^{2} \left( {r^{6} - 7r^{4} R^{2} + 29r^{2} R^{4} - 59R^{6} } \right) + \left( {r^{6} + r^{4} R^{2} + r^{2} R^{4} - 15R^{6} } \right)\zeta } \right)} \right\}} \right\}} \right], \hfill \\ \end{gathered} $$

35 $$ \begin{gathered} \overline{T}_{2} = \frac{{B_{m} \left( {r^{2} - R^{2} } \right)}}{{73728C_{1} C_{4} }}\left[ {2304C_{1}^{2} \left( {r^{2} + R^{2} } \right) + C_{2}^{2} M^{4} \left( { - 9r^{6} + 71r^{4} R^{2} - 181r^{2} R^{4} + 251R^{6} } \right)} \right. \hfill \\ \quad \quad \; - 64C_{1} \left\{ { - 2C_{2} M^{2} r^{4} + 7C_{2} M^{2} r^{2} R^{2} - 29C_{2} M^{2} R^{4} + 36dp/dz\left( {r^{2} + R^{2} } \right) + C_{3} G_{r} } \right. \hfill \\ \quad \quad \;\left. { \times \left( { - 2r^{4} + 7r^{2} R^{2} + 7R^{4} } \right) - 12r^{4} \zeta - 12r^{2} R^{2} \zeta - 12R^{4} \zeta } \right\} + 4\zeta \left\{ { - 128dp/dz\left( {r^{4} + r^{2} R^{2} + R^{4} } \right)} \right. \hfill \\ \quad \quad \; + \left. {C_{3} G_{r} \left( {9r^{6} - 23r^{4} R^{2} - 23r^{2} R^{4} - 23R^{6} } \right) + 18\left( {r^{6} + r^{4} R^{2} + r^{2} R^{4} + R^{6} } \right)\zeta } \right\} \hfill \\ \quad \quad \; + C_{2} M^{2} \left\{ { - 128dp/dz\left( {2r^{4} - 7r^{2} R^{2} + 11R^{4} } \right) + C_{3} G_{r} \left( {9r^{6} - 71r^{4} R^{2} + 181r^{2} R^{4} - 251R^{6} } \right)} \right. \hfill \\ \quad \quad \;\left. {\left. { + 6\left( { - 3r^{6} + 13r^{4} R^{2} + r^{2} R^{4} + 49R^{6} } \right)\zeta } \right\}} \right], \hfill \\ \end{gathered} $$

The approximate series solutions can be written as

36 $$ w = \mathop {\lim }\limits_{\varepsilon \to 1} \overline{w} = \overline{w}_{0} + \overline{w}_{1} + \overline{w}_{2} + \ldots , $$

37 $$ T = \mathop {\lim }\limits_{\varepsilon \to 1} \overline{T} = \overline{T}_{0} + \overline{T}_{1} + \overline{T}_{2} + \ldots , $$

The flux (*Q*) can be computed with the help of following expression

38 $$ Q = 2\mathop \smallint \limits_{0}^{R} ru\left( {r,z} \right){\text{d}}r. $$

The expression of impedance can be computed utilizing the above expression (38), thus, we have

39 $$ I_{m} = \frac{1}{Q}\int\limits_{0}^{L} {\left( { - \frac{dp}{{dz}}} \right)} {\text{d}} z. $$

## Significance of the study

In this section, we are going to investigate the plotted graphs for the most important physical variables versus the parameters of interest. Results have been computed on the basis of the following parametric values: ${G}_{r}=5,\zeta =1,{B}_{m}=5,\delta =0.3,{\Phi }_{1}=0.1,{\Phi }_{1}=0.15$ and $M=0.5$.

In addition, Table 1 presents the thermo-physical values of blood, diamond and silica for the numerical computations. The analytical and numerical results are performed based on the symbolic computational software, Mathematica.

**Table 1:** Thermo-physical features of blood, diamond and silica^30^.

	Blood	Diamond (D)	Silica (SiO_2_)
$\rho \left( {{\text{kg}}/{\text{m}}^{3} } \right)$	3617	3100	2650
$\kappa \left( {{\text{W}}/{\text{mK}}} \right)$	0.52	1000	1.5
$C_{p} \left( {{\text{J}}/{\text{kgK}}} \right)$	1050	516	730
$\sigma \left( {{\text{S}}/{\text{m}}} \right)$	1.33	34.84 × $10^{6}$	0.0005

### Velocity mechanism

**Figure 2:**
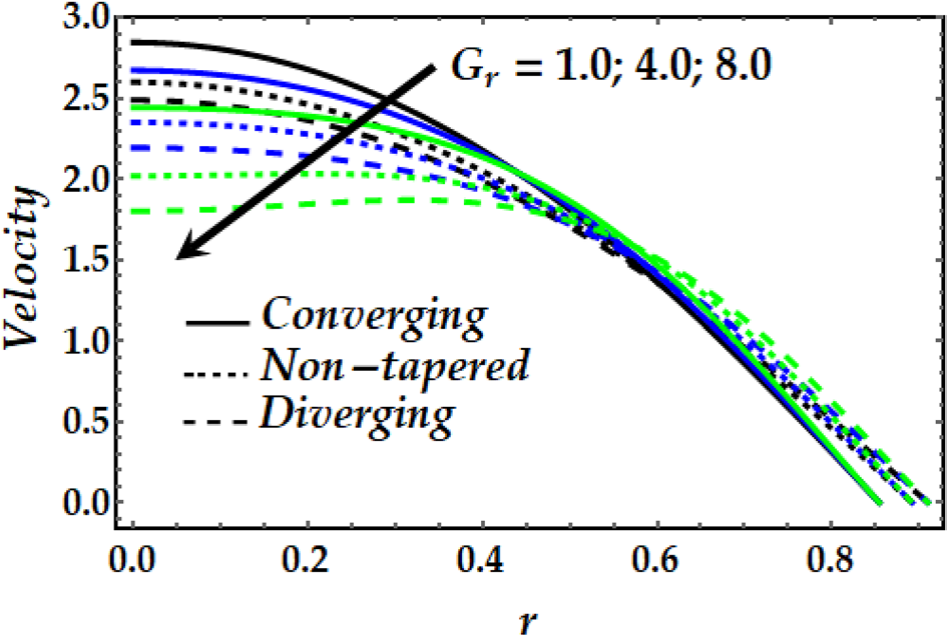
Velocity profile against distinct values of $G_{r} .$

**Figure 3:**
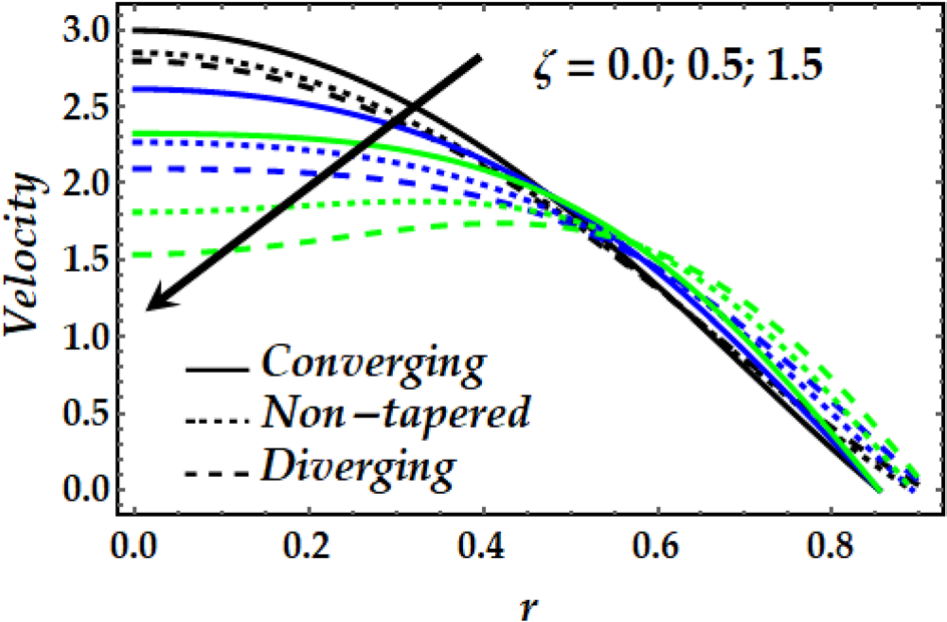
Velocity profile against distinct values of $\zeta .$

**Figure 4:**
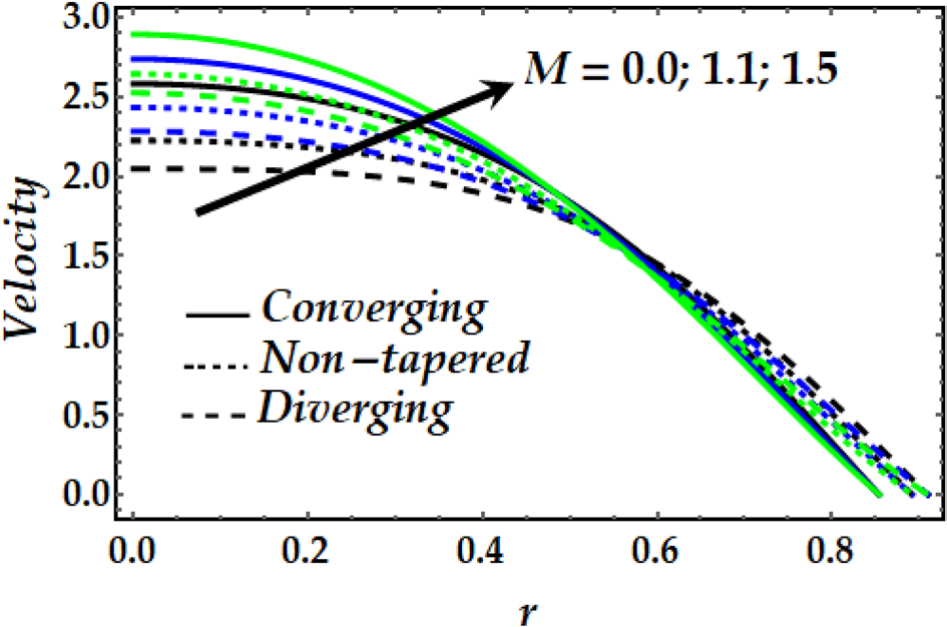
Velocity profile against distinct values of $M.$

**Figure 5:**
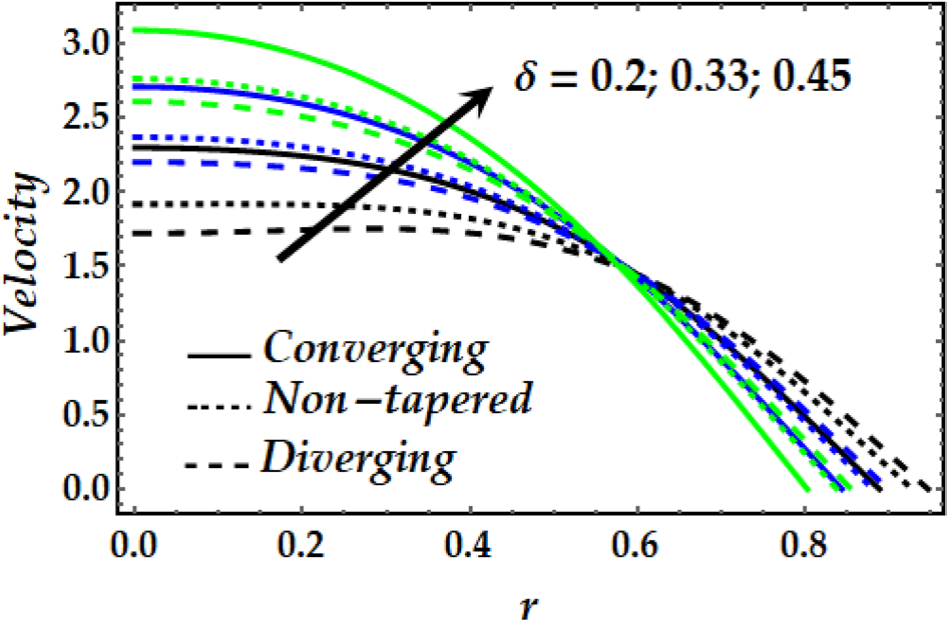
Velocity profile against distinct values of $\delta .$

**Figure 6:**
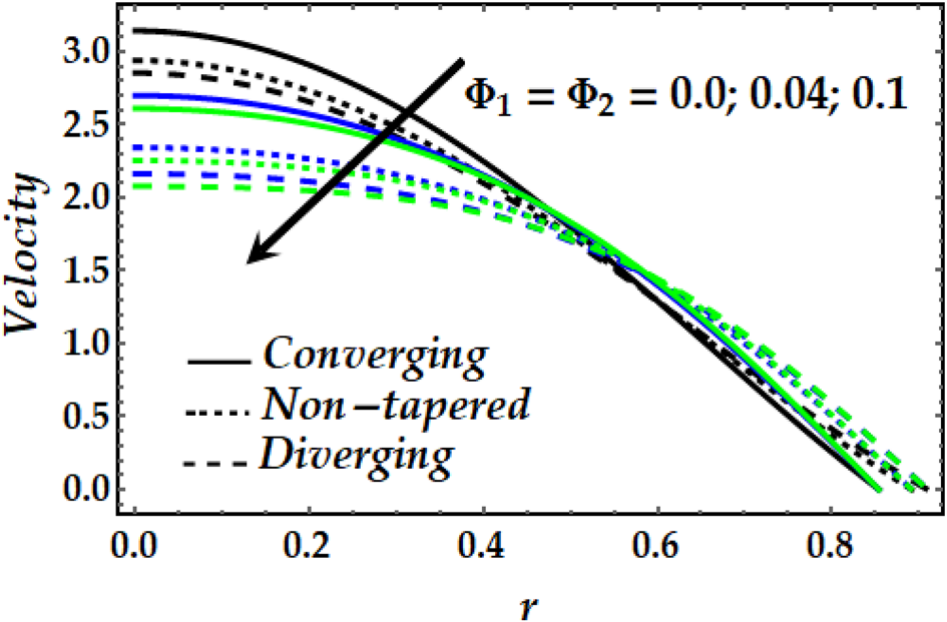
Velocity profile against distinct values of $\Phi_{1} ,\Phi_{2}$.

Figures 2, 3, 4, 5 and 6 reveal the dependency of the velocity distribution on the thermal Grashof number $Gr$, third-grade fluid parameter $\zeta$, magnetic parameter *M*, height of stenosis $\delta$ and nanoparticle volume fraction ${\Phi }_{1}$ and ${\Phi }_{2}$. Figure 2 represents the behaviour of velocity profile with various values of $Gr$ for converging, non-tapered and diverging arteries. It is noticed that $Gr$ reduces the flow greatly until *r* = 0.5 before the behaviour is reversed with an increase in $Gr$. Such reduction in flow velocity might be attributed to Lorentz force that opposes the flow. It is seen that for the converging artery, the flow is accelerated more than that of the non-tapered and diverging ones until *r* = 0.5 before the difference does not seem to be very effective after that for progressive values of $Gr$. Figure 3 elucidates the behaviour of velocity distribution with higher values of $\zeta$. It is depicted that $\zeta$ has the same effect of the flow field as does $Gr$. Figure 4 illustrates the impact of *M* on the velocity distribution along the different proposed conduits. It is observed that *M* accelerates the flow for various values of the pertinent parameters until *r* = 0.5 after which the flow is seen slightly decelerating for progressive values of *M*. Figure 5 presents the behaviour of the velocity profile along the various arteries for progressive values of $\delta$ where it is seen that $\delta$ has the exact same effect on the flow field as does *M*. Figure 6 illustrates the behaviour of the flow due to enhanced values of ${\Phi }_{1}$ and ${\Phi }_{2}$ where it is seen that the converging artery attains higher velocity distribution than that of the non-tapered and diverging ones until mid-artery. It is also observed that ${\Phi }_{1}$ and ${\Phi }_{2}$ causes a reduction in the flow velocity till almost midway of all the segments after which the effect is weakly increasing.

### Trapping mechanism

**Figure 7:**
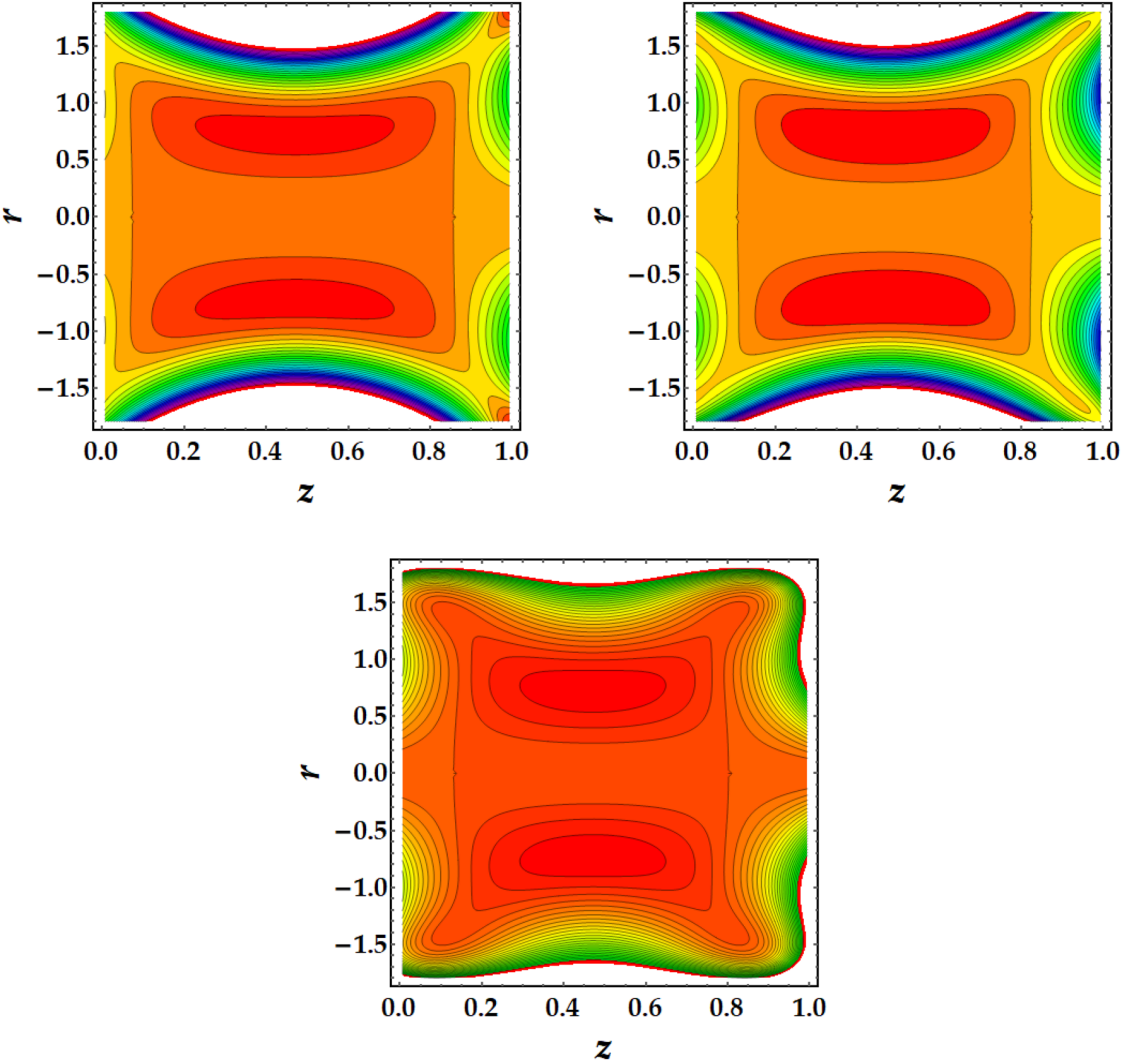
Behavior of streamlines against distinct values of (**a**) $\zeta = 0$, (**b**) $\zeta = 1$, (**c**) $\zeta = 2$.

**Figure 8:**
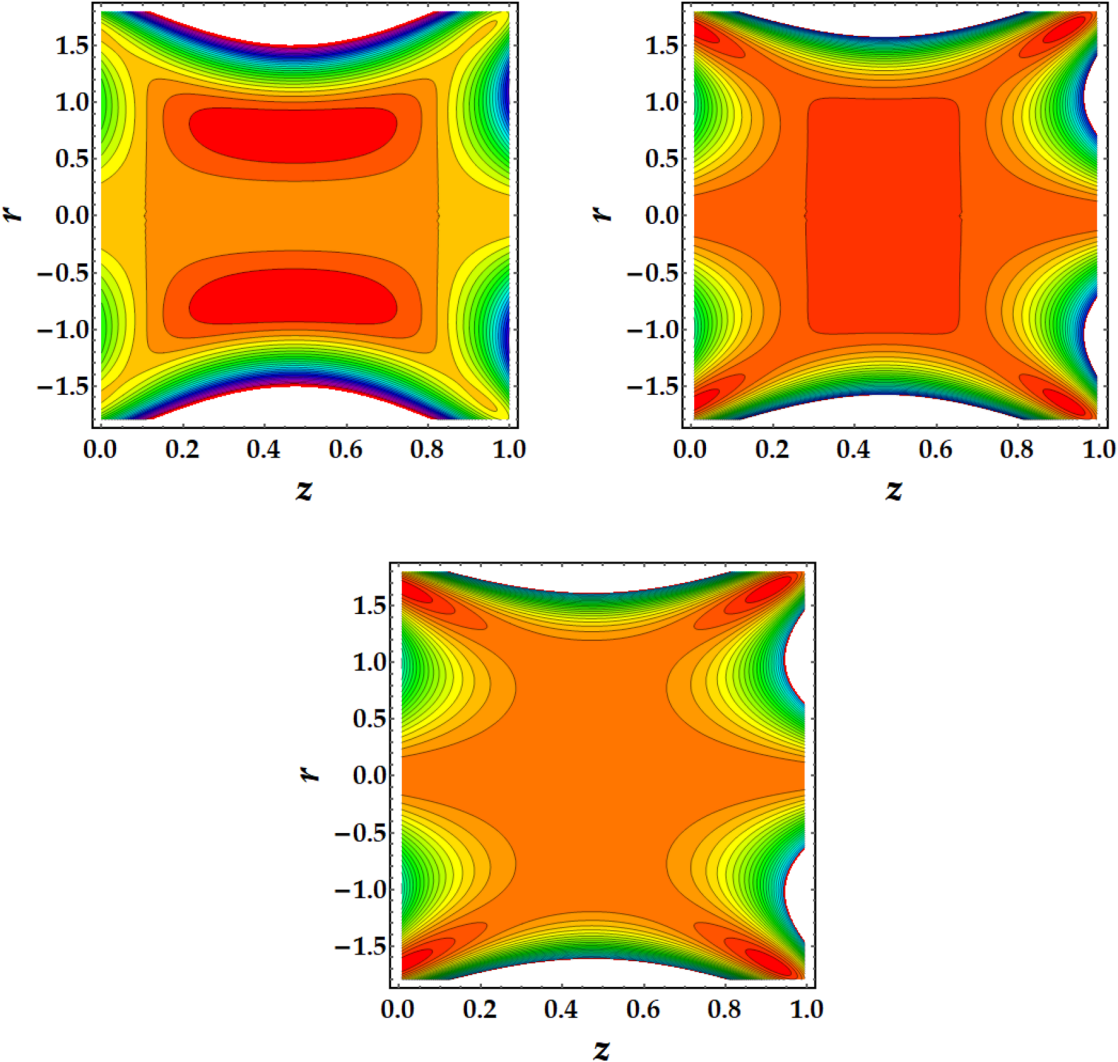
Behavior of streamlines against distinct values of (**a**) $G_{r} = 1$, (**b**) $G_{r} = 3$, (**c**) $G_{r} = 5$

**Figure 9:**
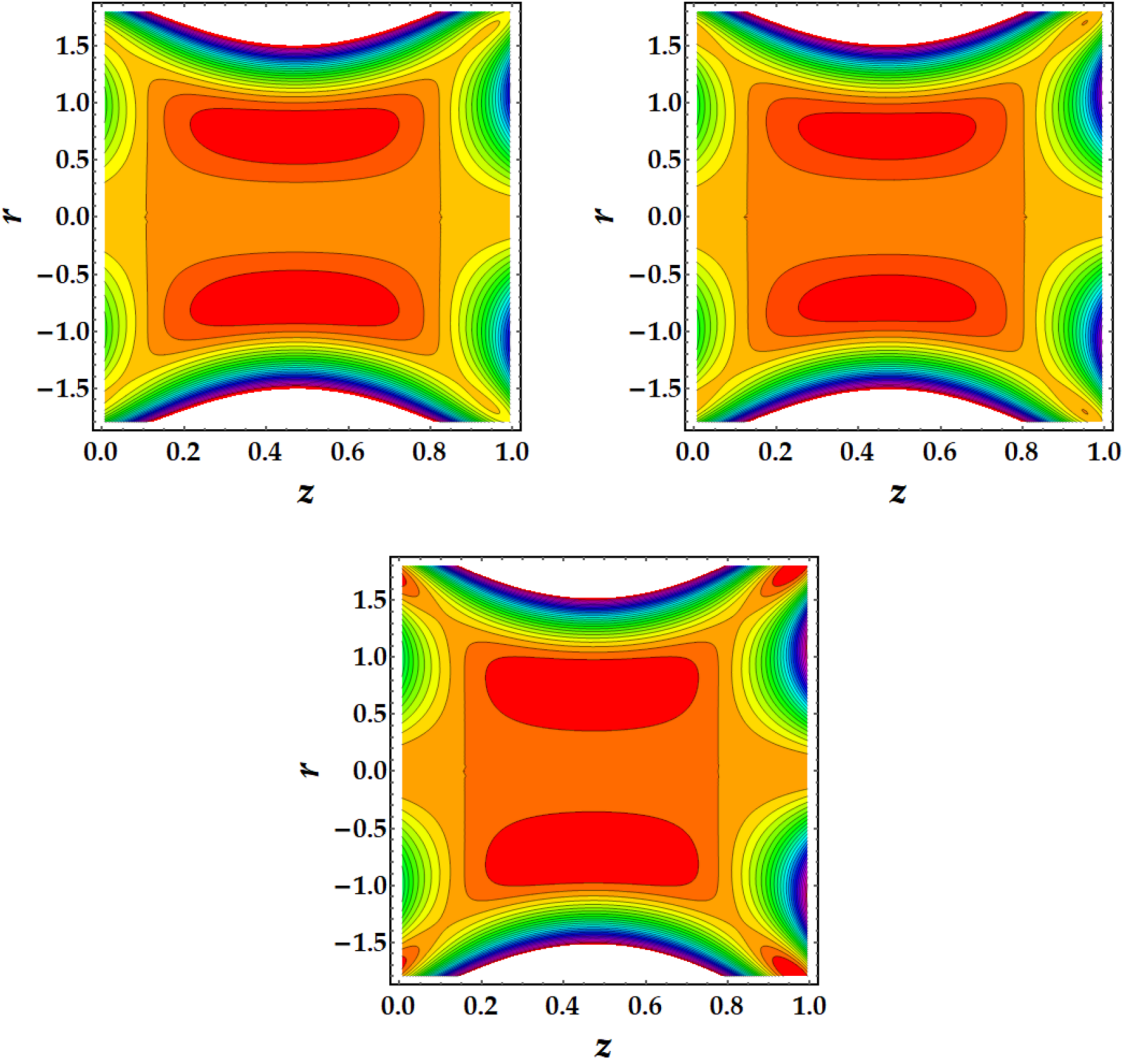
Behavior of streamlines against distinct values of (**a**) $M = 0.5$, (**b**) $M = 1$, (**c**) $M = 1.5$

**Figure 10:**
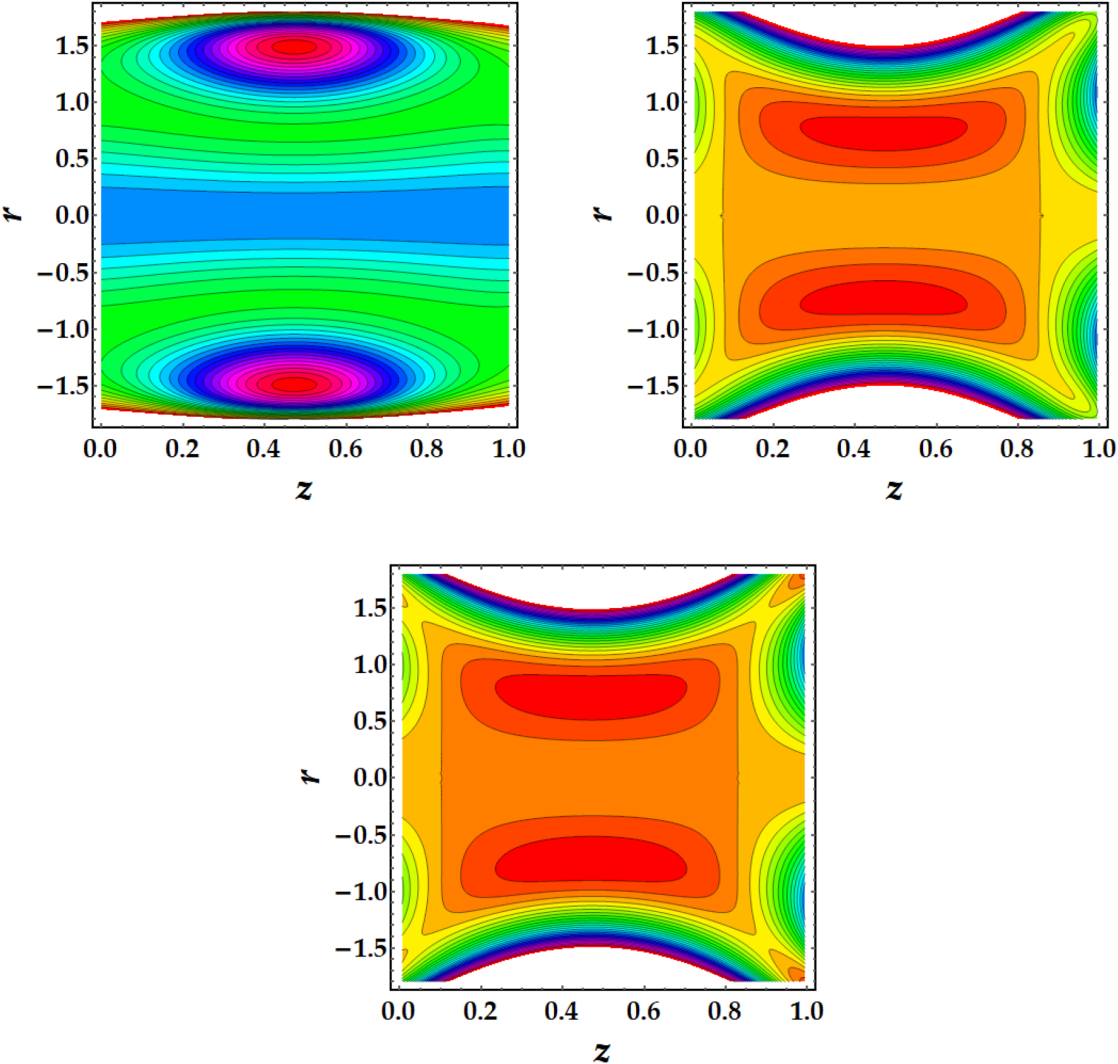
Behavior of streamlines against distinct values of (**a**) $\Phi_{1} = \Phi_{2} = 0,$ (**b**) $\Phi_{1} = \Phi_{2} = 0.1$, (**c**) $\Phi_{1} = \Phi_{2} = 0.2$.

Figures 7, 8, 9 and 10 are plotted to investigate the trapping as an important mehcanism in nanofluid flows that visualizes different trajectories. Ordinarily, trapping represents the development of inwardly circulating free eddy currents in the blood that are surrounded by streamlines. This mechanism is critical in biology because it assists in the development of blood clots and the harmful migration of bacteria. Figures 7, 8, 9 and 10 are plotted to illustrate the physical impact of pertinent parameters on the streamlines across the flow. It is seen from Fig. 7 that by increasing the third-grade fluid parameter $\zeta ,$ the number of trapped boluses increases while their size decreases. Figure 8 elucidates the impact of $Gr$ on the trapped region. Results show a drastic change in the shape and size of trapped region with an increase in $Gr$. The circulated region decreases then vanishes with progressive values of $Gr$. Figure 9 illustrates the effect of *M* on the streamlines with different values of parameters under consideration. It is notied that increasing *M* implies to a significant reduction in the number of circulating streamlines. It is shown from Fig. 10 that the impact of ${\Phi }_{1}$ and ${\Phi }_{2}$ is to decrease the number of circulating streamlines greatly along with increasing the size of trapped bolus to a great extent.

### Temperature profile

**Figure 11:**
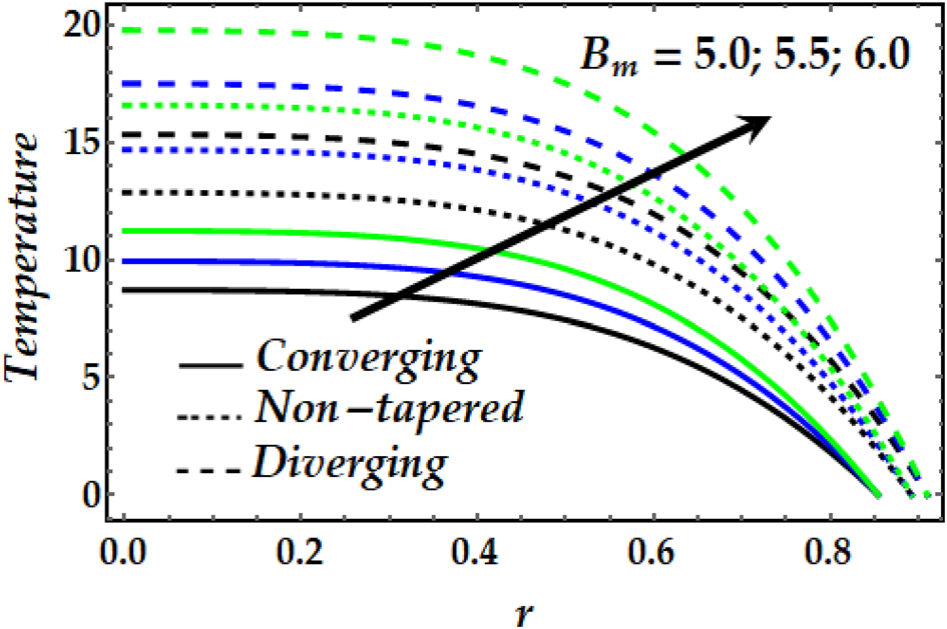
Temperature profile against distinct values of $B_{m} .$

**Figure 12:**
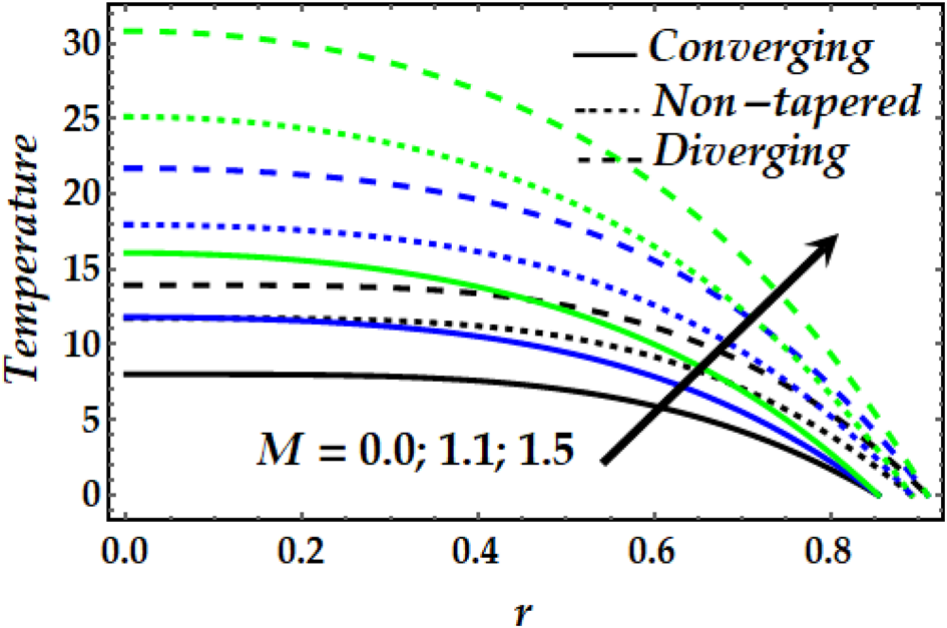
Temperature profile against distinct values of $M.$

**Figure 13:**
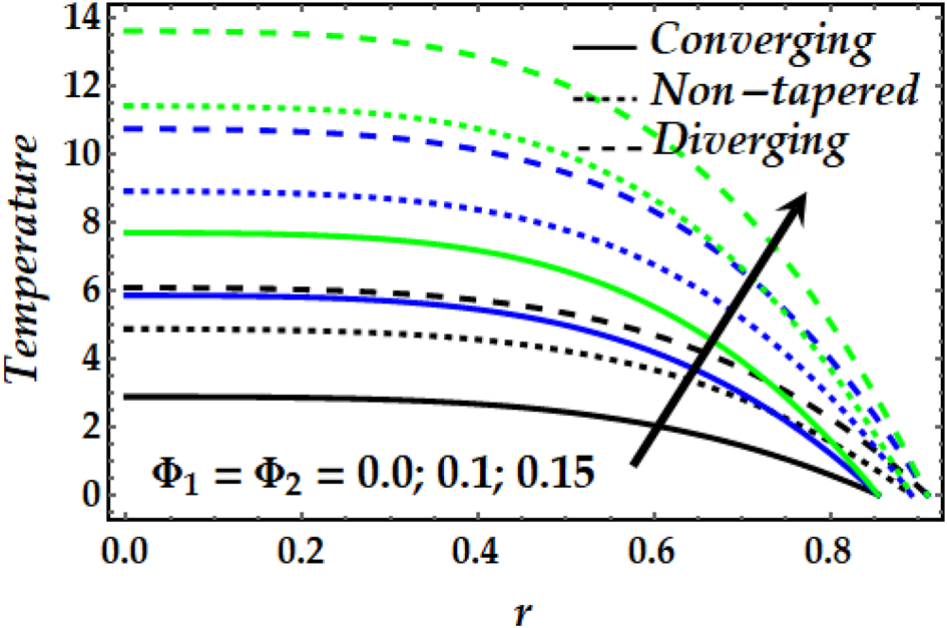
Temperature profile against distinct values of $\Phi_{1} ,\Phi_{2} .$

**Figure 14:**
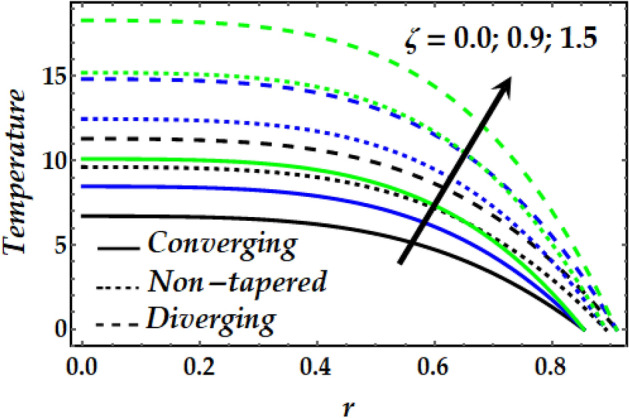
Temperature profile against distinct values of $\zeta .$

Figures 11, 12, 13 and 14 depict the behaviour of the temperature distribution against the governing flow parameters. Figure 11 observes the effect of ${B}_{m}$ on the temperature profile for the converging, non-tapered and diverging arteries. It is noticed that ${B}_{m}$ has an increasing effect on the flow temperature. It is also seen that the temperature of the converging artery attains smaller values than that of the non-tapered and diverging arteries. Figure 12 is plotted to describe the behaviour of temperature with increasing *M* where is seen that the temperature increases steadily with an increase in M across the flow field for all arteries. Figures 13 and 14 illustrate the behaviour of the temperature profile for all arteries against enhanced values ${\Phi }_{1}$, ${\Phi }_{2}$ and $\zeta$. It is observed from both figures that ${\Phi }_{1}$, ${\Phi }_{2}$ and $\zeta$ enhance the temperature distribution across the flow greatly. It is also noticed that the diverging artery has the highest temperature profile across all arteries. Further, it is seen from Fig. 14 that the temperature of Newtonian fluid is smaller than that if non-Newtonian one across the arteries.

### Impedance profile

**Figure 15:**
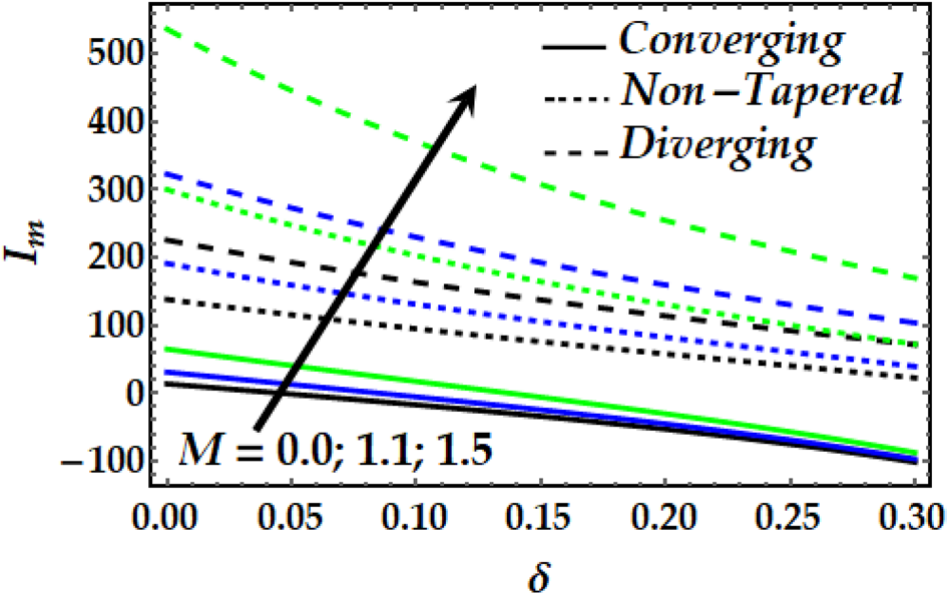
Impedance profile against distinct values of $M.$

**Figure 16:**
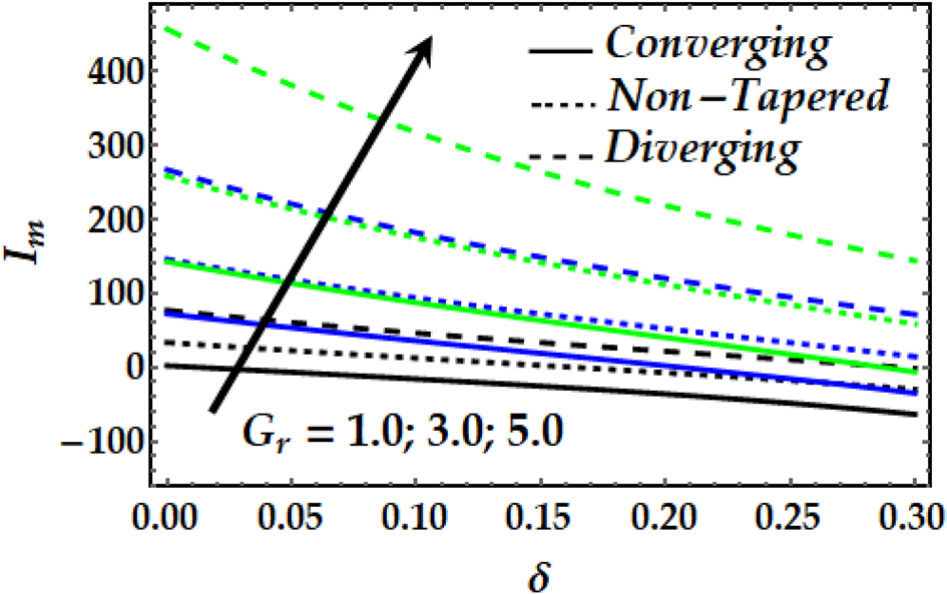
Impedance profile against distinct values of $G_{r} .$

**Figure 17:**
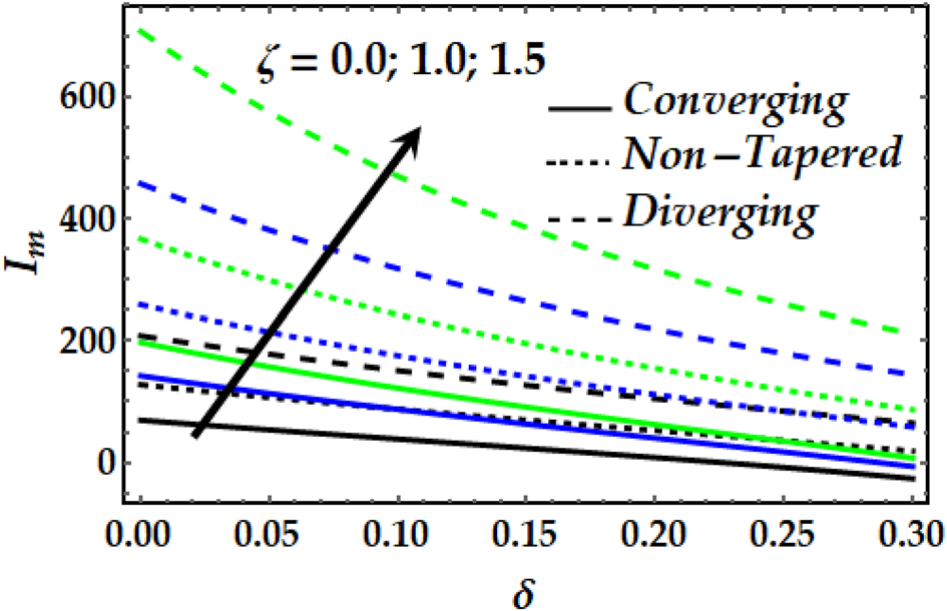
Impedance profile against distinct values of $\zeta .$

**Figure 18:**
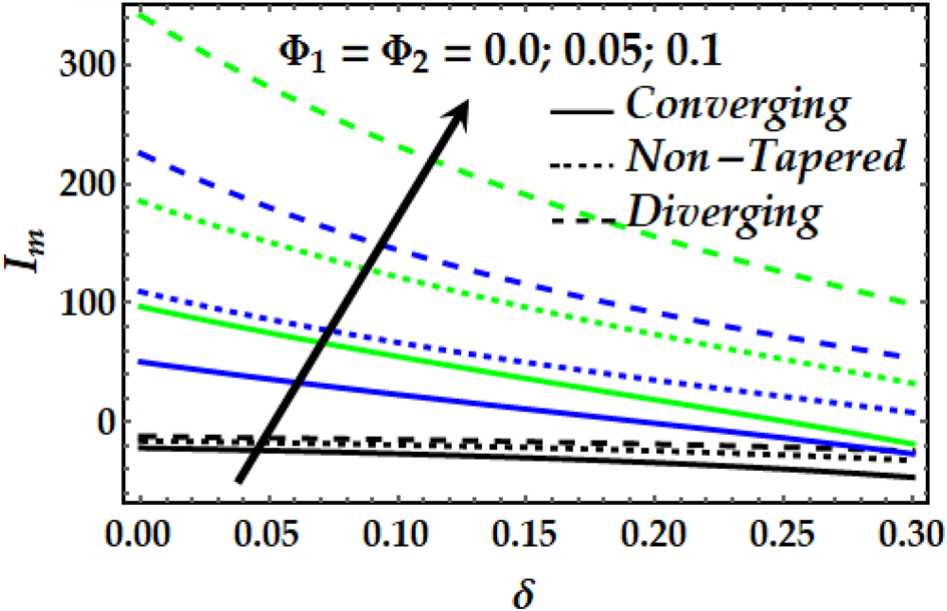
Impedance profile against distinct values of $\Phi_{1} ,\Phi_{2} .$

Figures 15, 16, 17 and 18 are plotted to present the effect of *M*, *Gr*, $\zeta$, ${\Phi }_{1}$ and ${\Phi }_{2}$ on the impedance *I*_*m*_ that is plotted against $\delta$ for various values of the pertinent parameters. Figure 15 shows that the impedance profile is greatly enhanced with progressive values of $\zeta$. It is noticed from Fig. 16 that *Gr* has the exact same effect on *I*_*m*_ as does $\zeta$. It is generally noticed that with both *M* and *Gr*, the converging artery’s impedance attains the least values among the non-tapered and diverging ones. Figures 17 and 18 describe the behaviour of *I*_*m*_ with $\zeta$, ${\Phi }_{1}$ and ${\Phi }_{2}$ where it is seen that these parameters boost *I*_*m*_ to a great extent for any value for the other parameters under consideration. It is also shown that the converging artery has the least impedance than do the non-tapered and diverging arteries. Further, it is seen from Fig. 17 that the impedance of Newtonian fluid is smaller than that of the non-Newtonian one.

### Wall shear stress

**Figure 19:**
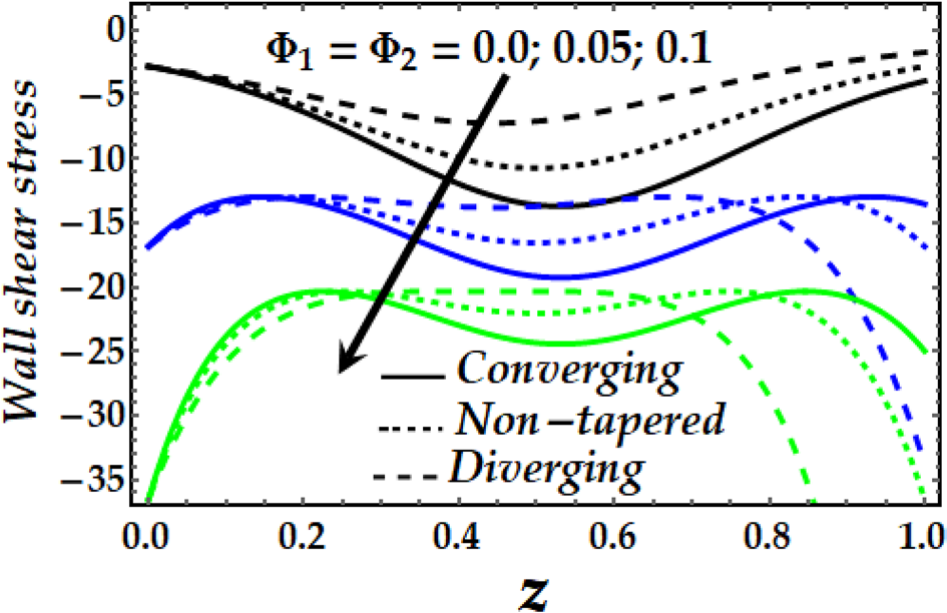
Wall shear stress against distinct values of $\Phi_{1} ,\Phi_{2} .$

**Figure 20:**
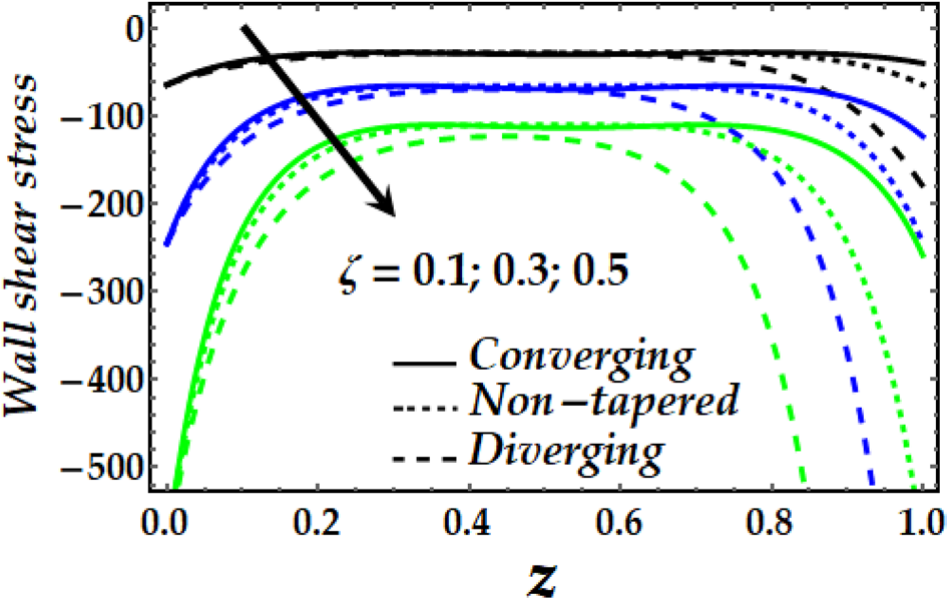
Wall shear stress against distinct values of $\zeta .$

**Figure 21:**
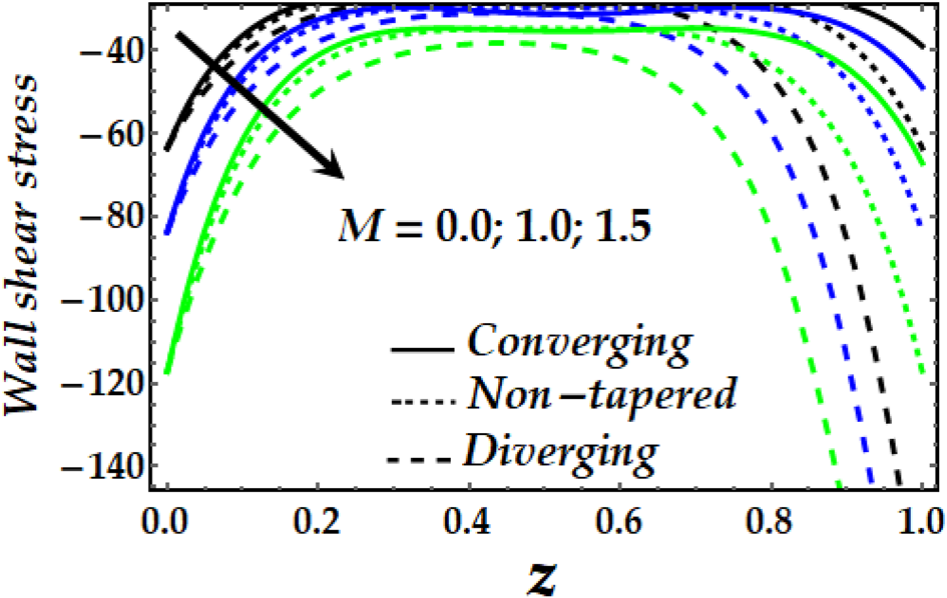
Wall shear stress against distinct values of $M.$

**Figure 22:**
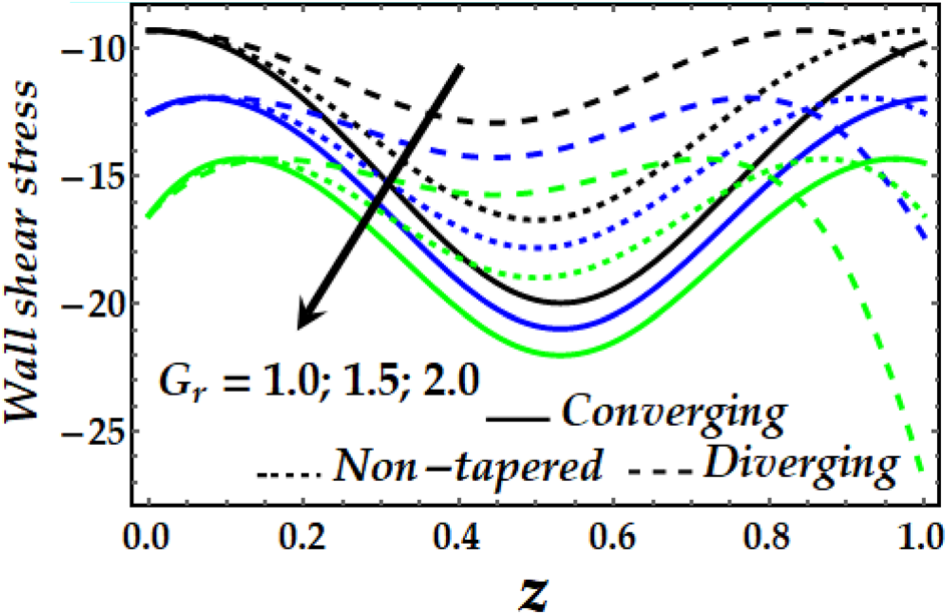
Wall shear stress against distinct values of $G_{r} .$

Figure 19, 20, 21 and 22 reveal the impact of ${\Phi }_{1}$, ${\Phi }_{2}$, $\zeta$, *M* and *Gr* on the wall shear stress with sundry values of the other parameters on interest. It is obvious that all the parameters’ impact on the wall shear stress is to reduce it steadily. It is also seen that the wall shear stress attains the highest values for the converging artery than that of the non-tapered and diverging ones with enhanced values of $\zeta$ and *M* as seen in Figs. 20 and 21. Inversely, Figs. 19 and 22 show that the wall shear stress attains the least values with increasing ${\Phi }_{1}$, ${\Phi }_{2}$ and *Gr* for any value of the other parameters of interest.

## Observations

In the proposed work, we aim to study the behavior of a hybrid nanofluid model with nano-diamonds and silica through a catheterized tapered artery with three distinct configurations: converging tapered, non-tapered and diverging tapered arteries. The third-grade fluid is used to represent the flow model. Magnetic field and with heat transfer are also taken into consideration and the governing system of equations is then solved in a closed form using the perturbation approach. The interpretation of the physical variables of interest is explained. The most important findings are as follows:

i. The flow decelerates with an increase of thermal Grashof number and this is attributed to Lorentz force.
ii. The volume fraction of nanoparticles causes a reduction in the flow velocity till almost midway of all the segments of the arteries.
iii. The magnetic parameter is seen to significantly reducing the number of circulating streamlines.
iv. The diverging artery has the highest temperature profile across all arteries.
v. The third-grade fluid parameter enhances the temperature distribution across the flow greatly.
vi. The converfing artery’s impedance attains the least values among the non-tapered and diverging ones.
vii. The wall shear stress increases steadily with an increase in the nanoparticles’ volume fraction, magnetic parameter, thermal Grashof number and third-grade parameter.
viii. The impedance and temperature of the Newtonian fluid are smaller than these of the non-Newtonian one.

## Data Availability

All data generated or analyzed during this study are included in this published article.
